# Overcoming water quality effects in biological monitoring: a case study of amphipod in situ exposures in Ontario agricultural streams

**DOI:** 10.1007/s10661-025-13665-8

**Published:** 2025-02-04

**Authors:** Matthew Hammond, Lisa Brown, John Struger, Lee Grapentine, Vince Palace, Adrienne J. Bartlett

**Affiliations:** 1 Aquatic Contaminants Research Division, Environment and Climate Change Canada, Burlington, ON Canada; 2 Water Quality Monitoring and Surveillance Division, Environment and Climate Change Canada, Burlington, ON Canada; 3 Watershed Hydrology and Ecology Research Division, Environment and Climate Change Canada, Burlington, ON Canada; 4 IISD-Experimental Lakes Area, Winnipeg, MB Canada; 5 Freshwater Institute, Fisheries and Oceans Canada, Winnipeg, MB Canada

**Keywords:** *Hyalella azteca*, Organophosphate pesticides, In situ exposures, Biomonitoring, Water quality, Seasonal

## Abstract

Bioindicators add valuable understanding of biological impacts to contaminant monitoring programs. However, attributing effects (e.g., mortality and growth impairment) to contaminant exposures is challenging because of potential confounding by environmental variables. We assessed the influence of four water quality variables (temperature, dissolved oxygen, pH, and conductivity) on contaminant effects assessments during in situ exposures of the amphipod *Hyalella azteca* in six agricultural and urban watersheds in southern Ontario, Canada (2005–06, 2008–10). We further tested whether sampling in specific months of the growing season would minimize confounding effects. While high toxicity from organophosphate and carbamate pesticides increased mortality and reduced growth in caged amphipods, warmer stream temperatures also affected endpoints, increasing mortality and improving growth. Seasonal patterns indicated early summer (June) as optimal for detecting impacts when: (1) stream pesticide concentrations were highest, (2) acetylcholinesterase (AChE) inhibition (a biomarker of organophosphate/carbamate exposure) was highest, and (3) stream temperature was below its seasonal peak. Specifically, higher correlations among organophosphate pesticide concentrations, AChE inhibition, and mortality indicated better attribution of cause in this month (r = 0.53—0.76, *p* < 0.05). Ability to discriminate between pesticide-impacted sites and reference sites was also greater than other months (June = 100% correct assignment as high-impact or reference site). Considering sampling times of stream bioindicators that maximize pesticide responses and limit confounding effects of water quality may improve the accuracy and resource-efficiency of biological monitoring programs.

**Supplementary Information:**

The online version contains supplementary material available at 10.1007/s10661-025-13665-8.

## Introduction

There is global concern regarding the impacts of agricultural pesticides on the environmental health of streams, rivers, and lakes (Mateo-Sagasta et al., 2017). Chemical monitoring programs, such as the pilot *National Water Monitoring Program for Pesticides* in Canada (Glozier et al., 2017; Struger et al., 2004, 2008, 2016), are fundamental to assessing risks from contaminants in aquatic ecosystems (see Gilliom, 2007 for comparable monitoring in the United States). In addition, biological monitoring of indicator organisms adds critical information on the potential ecological impacts (Lehtonen et al., 2014), from toxic effects of known pollutants to the impacts of multiple and emerging contaminants (e.g., Bertrand et al., 2018; Cunha et al., 2017).

The North American freshwater amphipod, *Hyalella azteca* (Saussure)*,* is a sensitive and widely used bioindicator species for which well-developed and standardized test methodology has been established (e.g., Environment and Climate Change Canada, 2017). Lab and field studies using *Hyalella* have reported biological risks posed by metals (e.g., Borgmann et al., 2005; Couillard et al., 2008), nanomaterials (e.g., Kuehr et al., 2021), pharmaceuticals (e.g., García-Medina et al., 2015), industrial additives (e.g., PFAS; Bartlett et al., 2021), and pesticides (e.g., Ali & Mulla, 1978; Moore et al., 2007). An important field application has been the in situ deployment of caged amphipods to assess responses of biota to environmental disturbances, initially for inorganic pollutants causing lake acidification (e.g., Grapentine & Rosenberg, 1992) and mining effluents (e.g., Borgmann et al., 2007), and later for organic pesticide mixtures in agricultural streams (e.g., Bartlett et al., 2016; Phillips et al., 2004).

In situ exposures of indicator organisms provide more realistic assessments of pesticide impacts on aquatic ecosystems than laboratory studies (Phillips et al., 2004). However, caged organisms respond to many biotic and abiotic factors of natural environments (Cooper, 1965), so it is challenging to directly link observed biological effects (e.g., low survival or growth) to pesticide exposure. Apical endpoints of growth and survival in *Hyalella*, for example, can be impacted by temperature (e.g., Sprague, 1963), pH (e.g., Pilgrim & Burt, 1993), dissolved oxygen (e.g., Irving et al., 2004), and various dissolved ions contributing to electrical conductivity (e.g., chloride; Bartlett et al., 2012). When environmental variables trigger stress or mortality in indicator organisms, they confound interpretation and can make an exposure concentration appear more toxic (e.g., Minguez et al., 2012). Conversely, they may obscure toxic effects when water conditions are more favourable to organisms than reference conditions (e.g., warmer temperature; Scherer et al., 2013). Biomarkers can associate endpoints to exposures if they signal a specific response to chemicals of interest (e.g., inhibited acetylcholinesterase (AChE) activity from organophosphate or carbamate pesticides). However, even biomarker responses may vary unexpectedly across sites and seasons (Berra et al., 2004; Vioque-Fernández et al., 2007). Overall, clearer attribution of toxic effects is needed to distinguish between in situ effects due to water quality and those that are due to contaminants (e.g., pesticides).

New approaches are needed that help isolate the effects of contaminants from covarying environmental stressors such as hypoxia and high/low temperatures. Statistical models may “correct” a response to exposure for environmental conditions (e.g., Coulaud et al., 2011; Javidmehr et al., 2015). It may also be possible to time field studies to avoid the most confounding water quality conditions and aid interpretation of in situ exposure studies. To our knowledge, there has not been a systematic evaluation of field data to optimize the deployment of *Hyalella azteca* and reduce the confounding effects of water quality in pesticide exposures.

We hypothesize that in situ* Hyalella* exposures will provide the clearest assessment of pesticide impacts in months where; (i) pesticide exposure is high, (ii) biomarker responses are high and (iii) the confounding influence of environmental variables on biomarker responses is low. If supported, this hypothesis could improve resource allocation for biomonitoring programs by reducing repeated cage deployment studies within a season (cf. Spycher et al., 2018).

Here we evaluate a “reduced seasonal coverage” approach (Spycher et al., 2018) where bioindicator monitoring is confined to proposed “optimal exposure windows”. We assessed its effectiveness for gauging pesticide risk by expanding and analyzing a dataset of *Hyalella* exposures in southern Ontario, Canada agricultural streams, first presented by Bartlett et al. (2016). In southern Ontario, stream pesticide mixtures include a range of insecticides, herbicides, and fungicides that are used across large swathes of agricultural land (e.g., the fruit-growing Niagara Region). A recent analysis for the region suggests that acute risks to aquatic biota are theoretically low (Desrochers et al., 2024), but while pesticide application rates and stream concentrations are relatively well-known and monitored (e.g., Farm & Food Care Ontario, 2015; Struger et al., 2016), the actual in-stream biological effects of pesticide mixtures are not.

We tested two hypotheses concerning the biological effects of pesticides using in situ exposures:

1. Seasonal water quality fluctuations hinder the ability of in situ exposures to resolve pesticide effects because they introduce extremes of confounding environmental conditions (e.g., overly warm or cool temperatures), and;
2. Limiting deployments to a specific month will reduce the confounding effects of water quality and result in stronger correlations between pesticides and endpoints and clearer differentiation between impacted and non-impacted sites.

## Methods

### Study sites

**Fig. 1:**
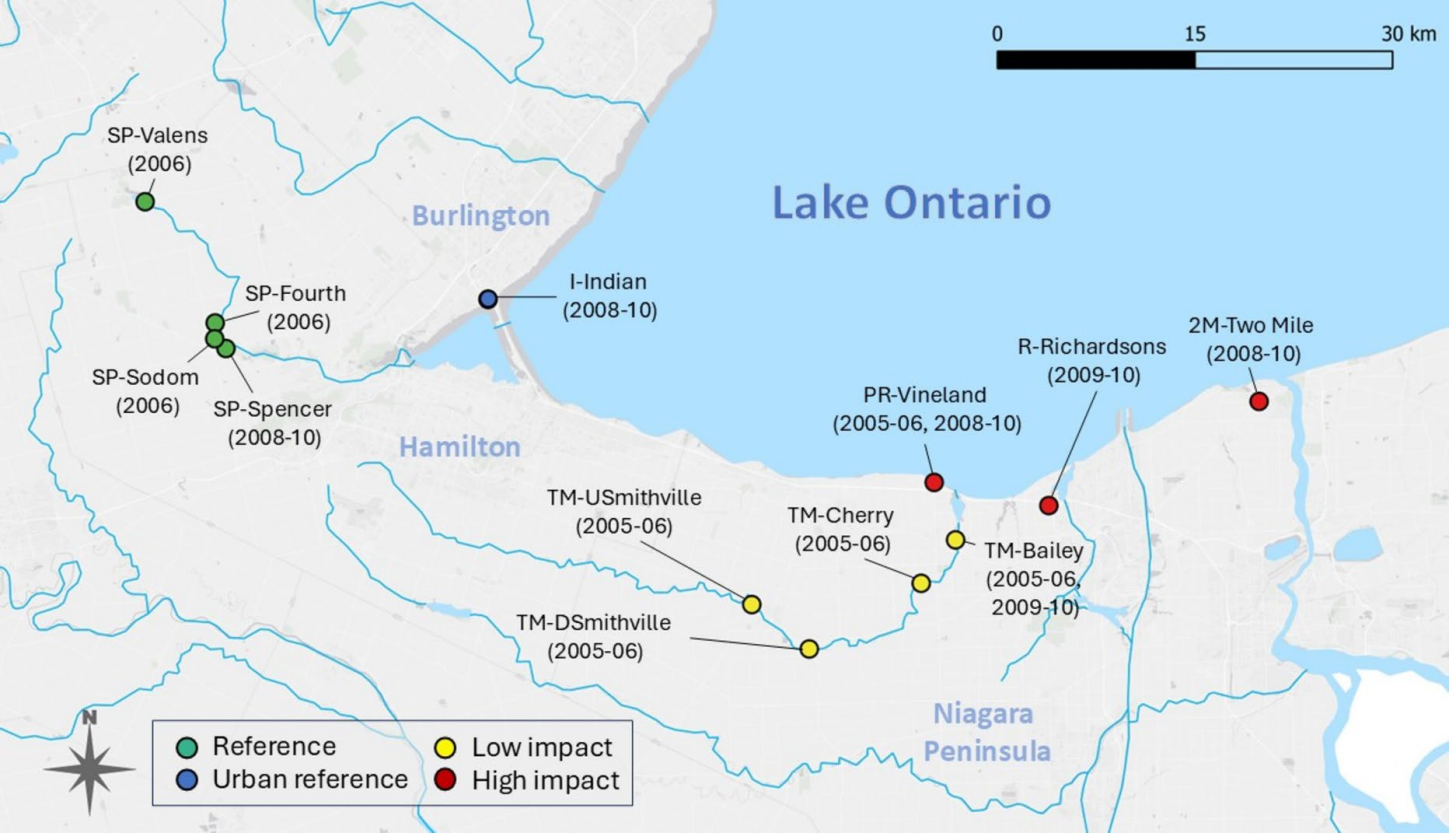
Sites in southern Ontario, Canada sampled for pesticides and in situ amphipod exposures, 2005–2010. Specific years sampled are indicated next to each site. See Table S1 for site details and land use descriptions. Prefixes denote watersheds containing sites: *I* – Indian Creek, *PR –* Prudhomme Creek, *R –* Richardson’s Creek, *SP* – Spencer Creek, *TM* – Twenty Mile Creek and *2 M* – Two Mile Creek

The present study extends the studies conducted by Bartlett et al. (2016), who in 2005–06 exposed *Hyalella azteca *in situ and measured pesticide residues at seven sites across three southern Ontario agricultural and mixed-use streams. Using the same methods, we conducted cage deployments for three additional years from 2008–10, adding 80 caging events to the original 2005–06 data and three new sites in additional watersheds (Fig. 1). Stream biological monitoring sites were originally chosen in 2003–04 based on the detection of organophosphate pesticides, with three reference sites added in 2006 (Bartlett et al., 2016).

Biological monitoring sites spanned a gradient of land uses, pesticide concentrations and, to some extent, years sampled. Monitored sites included agricultural stream locations in Ontario’s Niagara region of intensive fruit production, and reference and other mixed-use locations in adjacent regions (Fig. 1; see Table S1 for descriptions). Starting in 2008, a new reference site (SP-Spencer) was chosen from the same reference watershed (Spencer Creek) and an urban reference site (I-Indian) situated in Burlington, Ontario was added. Reference sites received low levels of pesticide inputs and were situated in areas of low agricultural intensity. Three sites in agriculture-dominated watersheds were characterized as ‘high-impact’ due to their high detected concentrations of organophosphates and carbamates. These were PR-Vineland, at which biological monitoring was conducted in all study years (2005–2006, 2008–10), and 2 M-Two Mile and R-Richardsons, which were added as sites in 2008 and 2009, respectively. Remaining ‘low-impact’ sites were within agricultural watersheds but had generally low concentrations of carbamates and organophosphates.

### Amphipod in situ exposures

Within each growing season, we aimed to deploy amphipods in study streams at minimum once during periods of pre-, peak- and post- typical pesticide application (April–May, June–August, and September–October, respectively). We conducted one-week in situ exposures of *Hyalella azteca* (clade 1; Major et al., 2013; Leung et al., 2016) at study sites according to methods originally developed by Borgmann et al. (2007) and further described in Bartlett et al. (2016). Briefly, adult amphipods (aged 5–13 weeks at deployment) of the same source population were cultured at Fisheries and Oceans Canada (Freshwater Institute, Winnipeg, Manitoba, Canada; 2005–2006) and Environment and Climate Change Canada (Canada Centre for Inland Waters, Burlington, Ontario, Canada; 2008–2010) and added to clear acrylic cages (8 cm long; *N* = 20/cage, except for July 2005 where *N* = 10) sealed at each end with 300-μm Nitex mesh. Five replicate cages containing cotton gauze and 15 mg of ground TetraMin (Ulrich Baensch, Melle, Germany) fish food flakes each were deployed at each site. Table S2 provides further details of in situ exposure frequencies and methodology across study sites.

Laboratory controls were maintained for each one-week caging period. As noted in Bartlett et al. (2016), procedures for laboratory controls changed in 2006, from being maintained at 25 °C under standard culturing conditions in 2005 to being maintained at approximate seasonal field temperatures and within cages held in 10-L buckets containing culture water from 2006 onwards. There was no difference in mortality (one way ANOVA, *p* > 0.05) or wet weight (*p* > 0.05) among years using the same or different control types. However, AChE activity in control animals differed among years (*p* < 0.01), specifically for 2005 when the original (i.e., standard culturing conditions) control type was used, and therefore 2005 data were not included in statistical analyses of AChE inhibition.

After cages were retrieved from the field sites, amphipods were transported back to the laboratory in buckets filled with site water, where they were counted, rinsed, blotted dry, weighed as a group (i.e., wet weight per cage), and transferred to a microcentrifuge tube kept at −80 °C. Mortality was defined as the percentage of initially added animals no longer surviving at the time of retrieval. Percent growth reduction was calculated relative to controls as:

1 $$\mathrm{Growth}\;\mathrm{reduction}\;(\%)=100\times\left({\overline{\mathrm X}}_{lab}-{\overline{\mathrm X}}_{\mathrm{ij}}\right)/{\overline{\mathrm X}}_{lab}$$

where ${\overline{\mathrm X}}_{ij}$ is the mean wet weight of caged amphipods at site *i* and caging period *j* and ${\overline{\mathrm X}}_{lab}$ is the mean wet weight of corresponding lab controls.

Two caging periods (September 2005, August 2008) had a mean mortality > 10% (12% and 11%, respectively) in lab controls. Data from these caging events were removed from analysis. Mean mortality of remaining controls was very low (mean = 2.5% ± 2.3 SD). Amphipods were transplanted into stream water that was, on average, within 3 °C of the temperature at which they were cultured, though our measurements did not encompass the full temperature range in streams. Still, there was no correlation between the temperature differential of culture and stream water and mortality (r = 0.15, *p* > 0.05), suggesting no systematic effect of different stream versus culturing temperatures.

### Pesticide sampling & toxicity assessment

Pesticides were sampled at caging sites as part of an Environment and Climate Change Canada (ECCC) pesticide monitoring program, active from 2002 to 2016. Grab samples were taken at sites approximately monthly throughout the agricultural growing season and coordinated with the deployment and retrieval of *Hyalella* cages. Between 2005 and 2010, water samples were analyzed by ECCC’s National Laboratory for Environmental Testing (Burlington, ON) for 65 pesticides including: (i) 13 organophosphate insecticides (azinphos methyl, chlorpyrifos, diazinon, dimethoate, disulfoton, ethion, fonofos, malathion, naled, parathion, phorate, phosmet, and terbufos) of primary interest for their high toxicity, (ii) 16 acid herbicides commonly found in agricultural mixtures (e.g., 2,4-dichlorophenoxyacetic acid [2,4-D], clopyralid), (iii) nine neutral herbicides (e.g., atrazine, metolachlor), (iv) six carbamate pesticides with similar mode of action to organophosphates (e.g., carbaryl, pirimicarb), measured only from 2008 onwards, and (v) 18 sulfonylurea pesticides and one fungicide (metalaxyl), also measured from 2008 onwards. Three neutral thiocarbamate herbicides (butylate, diallate, and triallate) were screened for but never detected. Organophosphate pesticides and acid/neutral herbicides in surface water samples were measured by gas chromatography/mass spectrometry using methods described by Bartlett et al. (2016). Sulfonylurea pesticide concentrations were measured by coupled liquid chromatography/mass spectrometry using methods described in Struger et al. (2011). Metalaxyl and carbamate/thiocarbamate concentrations were similarly quantified using methods of Struger et al. (2016). See Table S3 for a list of pesticides detected, method detection limits (MDLs) and their environmental concentrations.

We related pesticide concentrations to associated Aquatic Life Benchmarks (acute) for freshwater invertebrates developed by the United States Environmental Protection Agency (US EPA, 2023). The benchmarks are derived from LC50 data (usually 48- to 96-h tests) and typically correspond to the most sensitive species in a taxonomic group. Benchmarks were available for 80% of measured compounds which formed the basis of our analysis. We expressed the additive toxicity of compounds using the Pesticide Toxicity Index (PTI, specifically the “Sensitive PTI”) of Nowell et al. (2014). The PTI uses a summed Toxic Units approach formulated as:

2 $$\mathrm{PTI}={\sum }_{\mathrm{i}=1}^{\mathrm{n}}{\mathrm{E}}_{\mathrm{i}}/{\mathrm{TC}}_{\mathrm{i}}$$

where *E*_*i*_ is the environmental concentration of pesticide *i* and *TC*_*i*_ is its toxic concentration (i.e., the benchmark).

Prior to calculating the PTI at each caging site, we replaced concentrations that were below minimum detection limits (non-detects) with zeros. We deemed this approach more appropriate than replacing them with MDLs because in some cases MDLs exceeded benchmarks, leading to anomalously high toxicity values for reference sites. Replacing non-detects with zeros eliminates this effect but may also underestimate pesticide concentrations and toxicity estimates (de Solla et al., 2012). Our analysis therefore evaluates toxicity conservatively.

To assess the potential toxicity of different pesticide types, we calculated the PTI for five pesticide groupings (organophosphates, carbamates, acid herbicides, neutral herbicides, and other, a group of the sulfonylurea herbicides and the phenylamide fungicide, metalaxyl). Calculation was restricted to water samples taken during the one-week caging period or, in one case (August 2005) the week before cage deployment. Where multiple water samples were taken during the caging period, pesticide values were averaged to estimate one PTI value per site per caging period.

### Water quality variables

Temperature, dissolved oxygen, conductivity, and pH were measured at sites during each caging period. In years 2005, 2006, and 2008, we took measurements at the time of cage deployment and retrieval using handheld field probes. We converted a small number of 2005 observations where only oxygen saturation (%) was measured to mg/L using elevation and temperature data in an online calculation tool (University of Minnesota, 2015). In 2009 and 2010, we deployed a YSI 6600 Extended Deployment Sonde (model 6600DEDS-M) at six sites (TM-Bailey, I-Indian, PR-Vineland, R-Richardsons, SP-Spencer and 2 M-Two Mile) for hourly measurements of all water quality variables. To make these measurements comparable with earlier years, we used only the first value after deployment and last value prior to retrieval for statistical analysis. To obtain a single value for each caging event, we calculated the mean of deployment and retrieval values.

### Acetylcholinesterase activity & protein measurement

Caged amphipods were kept frozen at −80 °C for up to 22 months prior to acetylcholinesterase (AChE) activity and protein assays. Storage time had no significant effect on measured AChE activity per gram protein or tissue (r = 0.25, *p* > 0.05 and r = −0.26, *p* > 0.05, respectively). For each cage retrieved from a site (*N* = 5) and laboratory controls, we analyzed AChE activity and protein content using a random subset of five amphipods. However, 8% of caging events could not be assayed due to high mortality (i.e., low tissue availability). AChE activity and protein determination followed methods adapted for microplate use, as described by Bartlett et al. (2016). Specific activity for AChE, in mmol/min/g protein, was then calculated following Fairbrother et al. (1991) as:

3 $$\text{Specific activity}=\left(\mathrm{A}\times {\mathrm{Vol}}_{\mathrm{R}}\times 1000\right)/\left(\mathrm{E}\times \mathrm{PL}\times {\mathrm{Vol}}_{\mathrm{H}}\times \mathrm{PR}\right)$$

where A is the change in absorbance per minute, Vol_R_ is the reaction volume, E is the extinction coefficient for 5,5’-dithiobis[2-nitrobenzoic acid] chromogen buffer reagent, PL is the pathlength, Vol_H_ is the homogenate volume, and PR is protein concentration in the homogenate.

Each sample was analyzed in triplicate and was re-analyzed if the Coefficient of Variation of the three values exceeded 15%. Percent AChE inhibition was then calculated relative to laboratory controls as:

4 $$\mathrm{AChE}\;\mathrm{inhibition}\;(\%)=100\times\left({\overline{\mathrm Y}}_{lab}-{\overline{\mathrm Y}}_{ij}\right)/{\overline{\mathrm Y}}_{lab}$$

where ${\overline{\mathrm Y}}_{ij}$ is the mean specific activity of replicate cages at site *i* and caging period *j* and ${\overline{\mathrm Y}}_{lab}$ is the mean specific activity of corresponding lab controls.

Amphipod size can influence levels of AChE activity (Xuereb et al., 2009). To test for this bias, we correlated amphipod wet weight and % AChE inhibition but found no relationship (r = −0.11, *p* > 0.05), similar to Bartlett et al. (2016).

### Statistical analysis

We tested for relationships between toxicity (PTI) of a pesticide group (e.g., organophosphates) and % AChE inhibition, % growth reduction, and % mortality using General Linear Models. To evaluate the influence of water quality on biomarker and endpoint responses, we added mean temperature, dissolved oxygen, pH, and conductivity for a site/caging period as model terms. We pooled observations across sites and caging months to include the largest possible range of pesticide concentrations and environmental conditions. For select analyses, we focused on organophosphate pesticides which were measured throughout the study. Carbamates, in contrast, were only measured from 2008 onwards. Variance Inflation Factors for linear model terms were generally low (maximum = 2.5) and tolerance high (minimum = 0.55) except where we tested for statistical interactions where variance inflation is commonplace. To identify the most parsimonious of competing models, we calculated Aikaike’s Information Criterion (AIC) corrected for small samples.

We tested that parametric assumptions of normality were met using Kolmogorov-Smirnoff and Shapiro–Wilk tests. Where they were not met, pesticide or biological response data were fourth-root or square-root transformed to meet assumptions. These transformations performed better than typical arcsine and logit transformations, and were preferred over log transformations that require adding arbitrary constants to zero values. For two caging events (1.5% of total), we obtained a small negative mortality value (−1%)—likely due to an extra amphipod being added at cage deployment. Percent growth reduction and percent AChE inhibition also had numerous negative values being relative to control values. We therefore rescaled (normalized) the variables to between 0 and 100 to allow square- or fourth-root transformation for use in statistical models. Water quality variables were log-transformed where necessary.

We tested for univariate differences among sites using the Kruskal–Wallis Median Test since homogeneity of variances assumptions were not always met based on Levene’s test. We used Discriminant Analysis to test whether sites weakly and strongly impacted by pesticides could be differentiated based on endpoint responses, after transforming and visually assessing data for multivariate normality. For these comparisons, we used data from individual, replicate cages (as opposed to means) to increase statistical power. All statistical tests used Statistica v.8.0 (Statsoft, Inc., 2007).

## Results

### Pesticide toxicity in Ontario study streams

Thirty-eight pesticides with toxicity benchmarks were detected at least once in study streams (Table S3). Acid and neutral herbicides were frequently detected (36% and 44% of samples on average), but with very low potential toxicity (mean PTI = 1.4 × 10^–5^ and 8.0 × 10^–4^, respectively). Sulfonylurea pesticides and metalaxyl, found in 18% of samples, had similarly low potential toxicity (mean PTI = 6.9 × 10^–4^). Carbamates had a higher PTI (mean = 0.01 ± 0.03) while occurring in 32% of water samples. Organophosphate pesticides, on the other hand, were infrequently detected (7% of samples) but had high toxic potential (mean PTI = 1.61 ± 4.38). Carbaryl was the only carbamate exceeding US EPA Aquatic Life Benchmarks in water samples, while four organophosphates did so (azinphos methyl, chlorpyrifos, diazinon, and malathion).

For a subset of caging periods where all pesticide groups were measured (44% of total), pesticide toxicities (PTI) of organophosphates and carbamates were significant predictors of mortality (Table 1; Table S4). Growth reduction was positively related to the PTI of carbamates, and negatively related to the PTI of neutral herbicides. The latter may be a spurious result because there is no known beneficial effect of neutral herbicides on growth, and stream concentrations were orders of magnitude below acute effect levels (maximum PTI = 0.02).

**Table 1:** Results of General Linear Models Predicting *Hyalella* Endpoints from Pesticide Toxicity Index (PTI) of Five Pesticide Groups. Only significant predictors are listed, with asterisks denoting significance level of model term: * p < 0.05; ** *p* < 0.01; *** *p* < 0.001. See Table S4 for full results

Endpoint/biomarker	Significant predictors	Semipartial correlation	R^2^_adj_	df	F	p
Mortality (%)^†^	Organophosphates*^†^	0.25	0.34	48	6.45	0.0001
	Carbamates**^†^	0.38				
AChE inhibition (%)^†^	-	-	0.10	45	2.13	0.079
Growth reduction (%)	Carbamates**	0.41	0.25	45	4.35	0.003
	Neutral herbicides**^†^	−0.35				

† Fourth-root transformed

### Influence of water quality variables on Hyalella endpoints

**Fig. 2:**
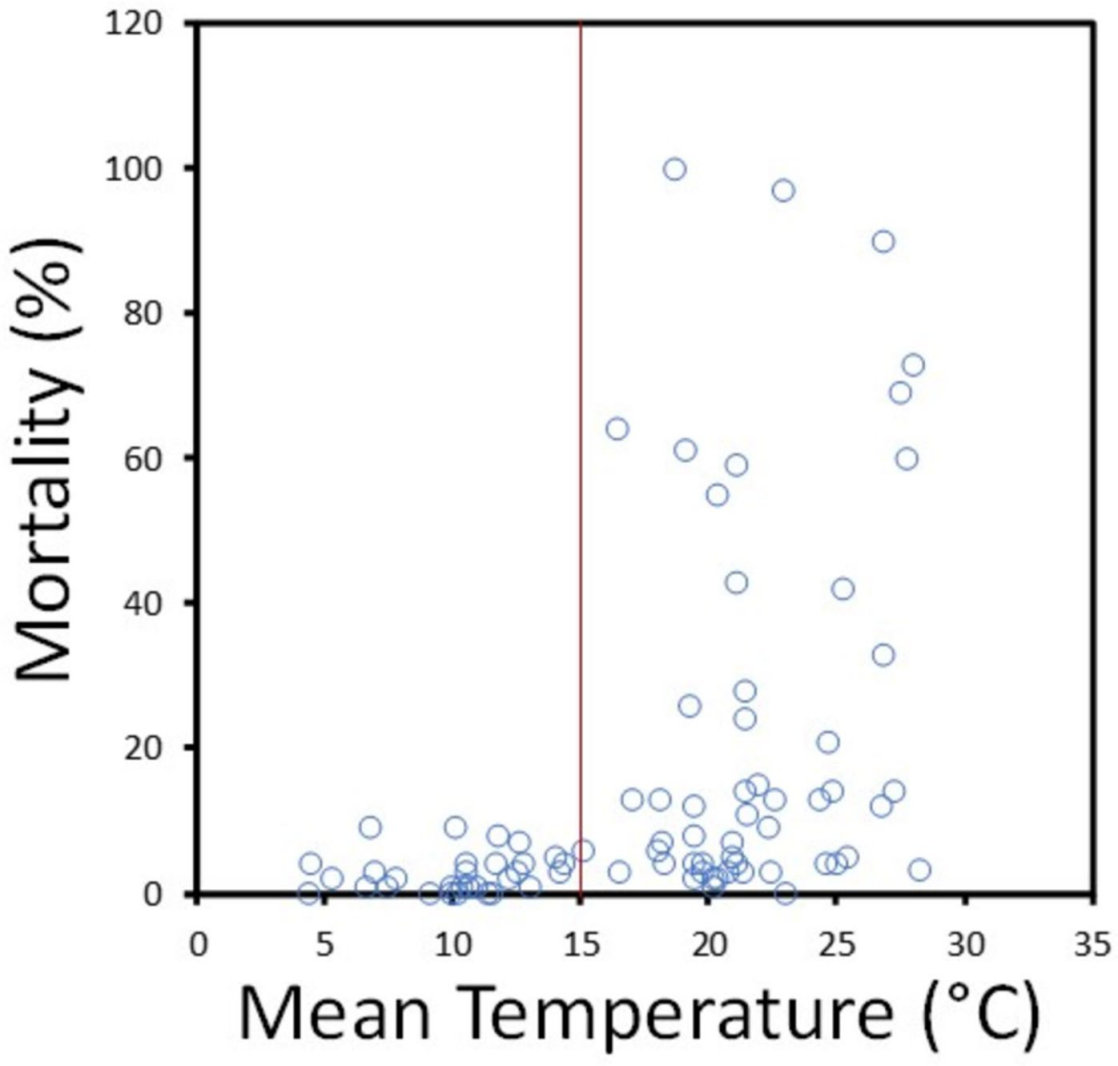
Mortality of caged *Hyalella* in Ontario study streams as a function of mean water temperature. To emphasize the effects of temperature, only in situ exposure periods with low toxicity from organophosphate pesticides (PTI < 1) are plotted. 94% of amphipod deaths occurred above 15 °C (red line)

Given high toxicity of organophosphates and their continuous measurement throughout the study, we focused on these pesticides to evaluate the influence of water quality variables. Linear models confirmed organophosphate PTI was a significant driver of AChE inhibition, mortality, and growth reduction (Table 2; see Table S5 for full results). Temperature was similar in its significance and magnitude of effect on *Hyalella* mortality (increasing it) and growth reduction (reducing it, i.e., increasing growth). Figure 2, for example, shows high mortality only above 15 °C when considering relatively unpolluted sites (PTI < 1). Adding an interaction term for organophosphate and temperature only marginally improved explanatory power (R^2^_adj_ = 0.49 vs 0.45 for mortality; 0.20 vs 0.15 for growth reduction; Table S6). Moreover, AIC values were lower (i.e., more parsimonious) for models without an interaction term (−133.72 vs −122.62 for mortality; 363.1 vs 386.3 for growth reduction).

**Table 2:** Results of General Linear Models Predicting *Hyalella* Responses to Organophosphate Pesticide Toxicity Index and Water Quality Variables. Only significant predictors are listed, with asterisks denoting significance level of model term: * *p* < 0.05; ** *p* < 0.01; *** *p* < 0.001. See Table S5 for full results

Endpoint/biomarker	Significant predictors	Semipartial correlation	R^2^_adj_	df	F	p
Mortality (%)^†^	Organophosphates***^†^	0.36	0.45	96	17.48	< 0.0001
	Mean temperature***^‡^	0.37				
AChE inhibition (%)^†^	Organophosphates***^†^	0.46	0.18	86	4.92	0.0005
Growth reduction (%)	Organophosphates*^†^	0.20	0.15	82	4.07	0.002
	Mean temperature**	−0.28				

^†^ Fourth-root transformed

^‡^ Log transformed

Including both organophosphates and carbamates in models restricted our analysis to the years 2008–2010, but showed additional significant toxic effects of carbamates on mortality and growth (Table S7). Organophosphates and carbamates both increased AChE inhibition. Dissolved oxygen and conductivity were also significant predictors in this reduced data set.

### Effect of reduced seasonal coverage

**Fig. 3:**
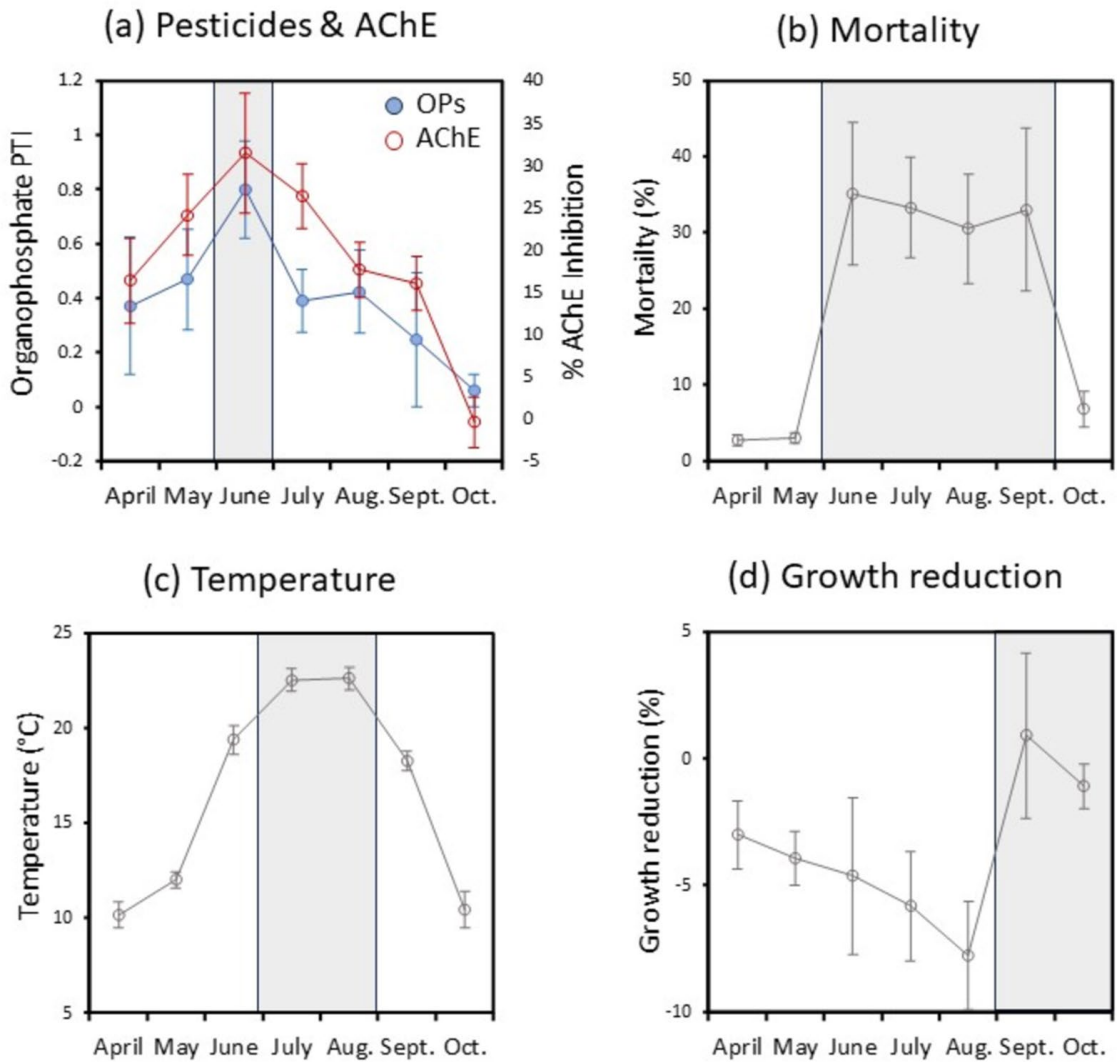
Seasonal variation of *Hyalella* responses and their drivers in Ontario study streams (2005–06, 2008–10). (**a**) Organophosphate toxicity (PTI) and AChE inhibition, (**b**) amphipod mortality, (**c**) mean stream temperature and (**d**) growth reduction relative to laboratory controls. Values are means ± standard error. Approximate peak months are indicated by shading

*Hyalella* endpoints and confounding water quality factors varied throughout the season. Organophosphate toxicity (PTI) and AChE inhibition both peaked in June, marking the month of maximum exposure and biomarker response (Fig. 3a). Mortality followed a similar pattern, peaking from June through September (Fig. 3b). Temperature, a key factor in mortality, peaked slightly later in July and August (Fig. 3c). Growth reduction, meanwhile, did not follow the seasonal pattern of pesticide exposure and peaked in September/October (Fig. 3d). In mid-summer months, caged amphipods grew larger than lab controls resulting in negative growth reduction, which is consistent with the confounding effect of warmer temperatures (Table 2).

We used the qualitative criteria for assessing maximum pesticide risk (see Introduction) to identify June as an optimal month for in situ exposures (see Table S8). The month had, on average, the highest organophosphate pesticide concentrations (Fig. 3a), the highest AChE biomarker response (Fig. 3a) and reduced influence of water quality variables (Fig. 3c). For the latter, while stream temperature was relatively warm in June (19.4 °C ± 3.5), it was 17% lower than the warmest month of August. June also had fewer warm water events exceeding 25 °C (20% of caging periods vs 31% and 24% for July and August, respectively).

**Fig. 4:**
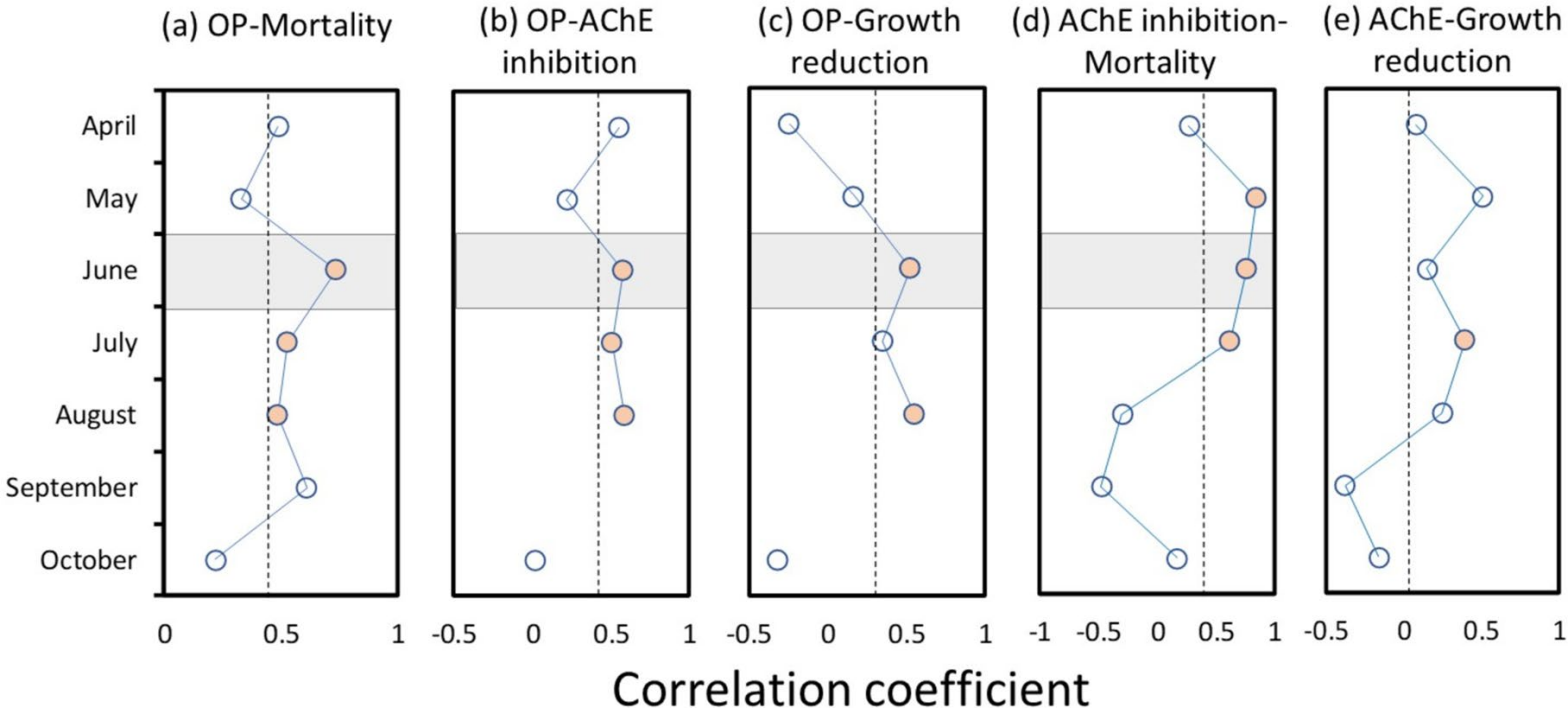
Correlation between organophosphate toxicity (PTI) and its biomarker, and *Hyalella* responses for different caging months. Organophosphate PTI was correlated with mortality (**a**), AChE inhibition (**b**), and growth reduction (**c**). AChE inhibition correlated with mortality (d) and growth reduction (**e**). Significant correlations (*p* < 0.05) are shaded orange. Dashed lines represent the overall correlations for all months combined. Gray shaded areas highlight strong and significant correlations occurring in June

We found the correlation between organophosphate PTI and mortality to be highest in June (Fig. 4a; r = 0.74, *p* < 0.001). June correlations were similarly strong for organophosphates versus AChE inhibition and growth reduction (r = 0.58, *p* = 0.019 and r = 0.53, *p* = 0.035, respectively) and were comparable to those seen in August (Fig. 4b-c). The AChE biomarker of organophosphate/carbamate exposure also correlated most positively with mortality in early summer (May, June; Fig. 4d), a pattern that was not as evident for growth reduction (Fig. 4e).

**Fig. 5:**
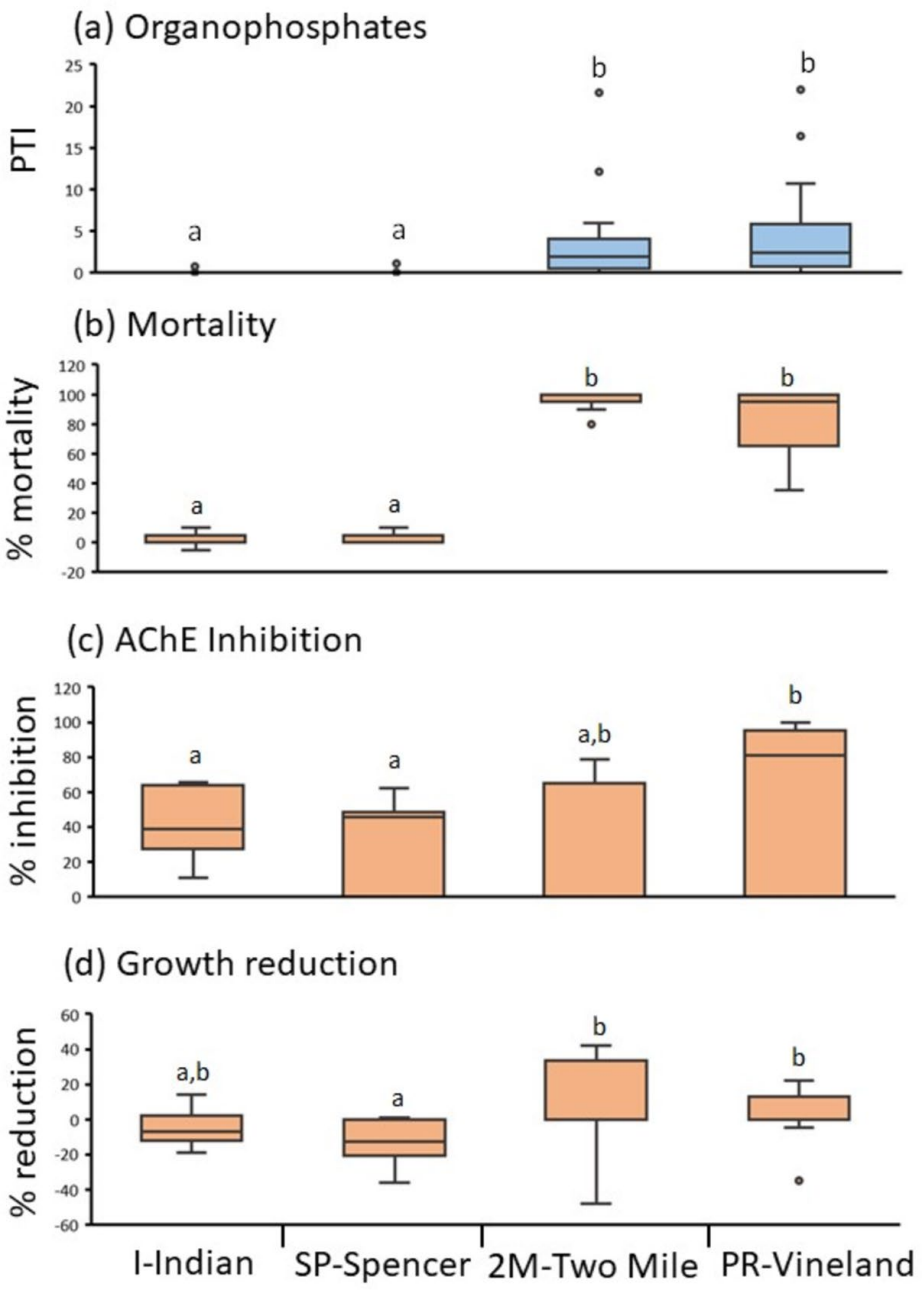
Boxplots comparing reference (I-Indian, SP-Spencer) and high-impact (2 M-Two-Mile, PR-Vineland) sites for (**a**) Pesticide Toxicity Index for organophosphates as blue bars, and for amphipod responses (orange bars) of (**b**) mortality, (**c**) AChE inhibition and (**d**) growth reduction, 2008–10. *a* and *b* denote groups indicated by post-hoc tests

The ability to discriminate among sites based on organophosphate impacts was also highest in June. We contrasted two reference sites (SP-Spencer and I-Indian) and two high-impact sites (P-Vineland and 2 M-Two-Mile) from 2008–10 when these sites were consistently sampled. Median organophosphate PTI differed among sites (χ^2^ = 32.17, *p* < 0.0001) and formed two groups – of reference and of high-impact sites – based on post-hoc tests (Fig. 5a). Mortality followed an identical grouping pattern (Fig. 5b), which other months did not (Table S9). AChE inhibition and growth reduction showed similar groupings to pesticide toxicity levels, but were not as strongly differentiated as mortality (Fig. 5c-d).

Discriminant analysis further showed a June peak for distinguishing impacted and reference sites based on amphipod responses (Table S10). In June, 100% of observations were correctly assigned to either a reference or high-impact category based on groups *a* and *b* in Fig. 5a (F = 50.95, *p* < 0.0001). In contrast, the next highest month was July (77.1% correct) with the lowest months in early growing season (April, May = 60.0% correct). June was also the only month in which mortality, AChE inhibition, and growth reduction were all significant variables in predicting reference or high-impact status.

## Discussion

Biological indicators are an important but resource-intensive addition to contaminant monitoring programs. Our findings suggest that in situ amphipod exposures can help assess risk with reduced effort if patterns of pesticide exposure and confounding by water quality are considered.

We found that water quality variables in Ontario study streams influenced the apical endpoints of mortality and growth, confounding their interpretation (Hypothesis 1). Water temperature, for instance, drove mortality to a similar degree as toxicity from organophosphate pesticides. Mortality in response to warmer summer conditions is common across taxa in aquatic environments (e.g., Landis et al., 2019) and has been documented in natural *Hyalella* populations (Cooper, 1965). Mortality during in situ exposures was associated with temperatures above 25 °C, which occurred in 14% of exposure periods. Sprague (1963) also reported significant mortality of laboratory *Hyalella* at temperatures exceeding 25 °C. Further potential for confounding by temperature was evident for growth, which was reduced by organophosphates but enhanced (less growth reduction) at warmer temperatures.

Indirect and multistressor mechanisms may also explain the links between temperature and mortality. Elevated temperature, for example, can increase invertebrate susceptibility to environmental stressors like low pH (Pilgrim & Burt, 1993) and pesticides (de Souza et al., 2023). Pesticide toxicity is also reportedly exacerbated in amphipods by high and fluctuating stream temperatures (Howe et al., 1994; Willming et al., 2013). We detected a positive interaction between organophosphate toxicity and temperature – which could indicate temperature enhancing the effects of pesticides or vice versa. However, including an interaction term did not meaningfully improve model fit based on AIC values. We therefore cannot be certain of the exact temperature mechanisms involved. Still, temperature can be concluded to add considerable variability to mortality as an endpoint. Our data (Fig. 3; Table 2) indeed suggest that temperature-related mortality may occur outside of periods of peak pesticide concentrations, and potentially be mistaken for pesticide-induced mortality if corroborating pesticide data are not available. Furthermore, any reductions in growth due to pesticides may be masked by the positive effect of water temperature (Table 2). While further study is needed, we speculate the improved growth relative to controls at higher temperatures may reflect higher amounts of edible detritus in streams and entering cages in peak summer months. Positive influences by confounding factors are seldom reported in the literature, but increase the chances of underestimating toxic effects when not accounted for (Scherer et al., 2013).

Inhibition of AChE is thought to provide a more targeted signal of exposure to organophosphates and carbamates. Adding to previous findings of biomarker variability (e.g., Day & Scott, 1990; Xuereb et al., 2009; Vioque-Fernández et al., 2007), we found only weak links between stream pesticides and suppressed AChE activity (R^2^ = 0.18). In our analysis of organophosphates, which included the most data, we found no evidence of AChE inhibition being influenced by water quality variables (see also Domingues et al., 2010). Rather, several other reasons, individually or in combination, may explain poor linkage between pesticide levels and AChE inhibition. First, several caging events in the most organophosphate-impacted streams had no AChE values due to low survival. We may therefore be missing instances of highest toxic inhibition. Second, infrequent grab samples of stream water are likely to omit some contaminant pulses, such as pesticide concentrations that can change by an order of magnitude or more around rain events (e.g., Leu et al., 2004; Petersen et al., 2012). Lastly, higher than expected AChE inhibition could arise from other compounds like unscreened pesticides, non-pesticides that may inhibit AChE (e.g., PAHs; Fu et al., 2018), or mixtures (e.g., organophosphates with atrazine; Anderson & Lydy, 2002).

The variability of *Hyalella* responses to pesticide exposure highlights the uncertainty inherent in bioindicators. Similar to Javidmehr et al. (2015), we caution against the use of in situ exposures without a reasonable accounting of water quality effects. In contrast to their statistical approach, however, we identify the potential for designing monitoring campaigns that provide more accurate signals by reducing or avoiding confounding effects.

### Efficient monitoring with reduced seasonal coverage

Our findings illustrate the ability to detect pesticide impacts with less sampling effort when the confounding effects of water quality are reduced (Hypothesis 2). Focused caging in June, when organophosphate toxicity peaked and confounding temperature impacts were lower than other months, demonstrated this in two ways. First, correlations between organophosphates and mortality/growth endpoints were highest in this month, providing statistical evidence that biological effects were due to pesticide exposure. Such attribution of cause is especially meaningful in stream environments where multiple stressors operate simultaneously. Second, we were best able to distinguish between low- and high-pesticide sites in June based on *Hyalella* endpoints and AChE inhibition. Targeted timing of caged exposures may therefore allow a particularly efficient assessment of ecological risk at a site. Effort may thus be focused on one or two influential months, as opposed to broader seasonal sampling.

It is important to note, however, that reduced seasonal coverage changes the aspects of biological risk detected. Focusing exposures on a month of high pesticide use and impact imparts the maximum risk (or close to it) facing stream organisms, analogous to sampling for pesticides during high flow events to detect peak concentrations (Bundschuh et al., 2014). We suggest that such monitoring is most appropriate where large, acute exposures may be expected to have long-lasting ecosystem impacts, such as mortality that resets invertebrate communities. The approach may be less relevant where cumulative exposures over a season are the main biological risk (e.g., chronic reproductive effects) or where high resolution data are required (e.g., monitoring for regulatory compliance). Second, Spycher et al. (2018) point out that reduced coverage approaches will necessarily miss pesticide peaks and their effects occurring outside of the chosen window (e.g., a large April chlorpyrifos peak in Two Mile Creek at 2 M-Two Mile). Similarly, choosing to deploy cages when pesticide use is highest on average for a region may not capture the highest month for a given site. Ability to resolve the exact magnitude and timing of pesticide risk at a site is therefore traded off for more efficient sampling. For this reason, we advocate the approach for basic or exploratory ecotoxicological monitoring when resources are limited.

### Agricultural stream monitoring: Ontario and beyond

Applying our “maximum risk, minimum confounding” approach offered insights into Ontario stream health that can be replicated elsewhere. In the most impacted streams (e.g., Prudhomme and Two-Mile Creeks), high toxicity came mainly from organophosphate pesticides characteristic of fruit and row crop production found in those watersheds. However, since the last in situ exposures in 2010, all four of the benchmark-exceeding organophosphates have been either discontinued/phased out (azinphos methyl, chlorpyrifos) or restricted in use in Canada (diazinon, malathion; Health Canada, 2023). Application rates of traditionally-used insecticides have indeed declined in recent years (Farm & Food Care Ontario, 2015), though have likely been replaced by newer products (e.g., neonicotinoids; Struger et al., 2017). Still, the acute risks of pesticide exposures across southern Ontario were estimated to be low as recently as 2016 (Desrochers et al., 2024). Ecological risks to invertebrates and other taxa may therefore have lessened if carbamates, which we found to have little association with toxicity in our caged exposures, or other alternatives have replaced organophosphates. Future monitoring is needed to confirm improvement in these waterways from what may be a relative peak of organophosphate impacts. The Ontario experience underscores the high risk to agricultural stream ecosystems for jurisdictions with more liberal organophosphate use (e.g., United States; Donley, 2019).

We suggest that a reduced coverage approach could be useful in other jurisdictions where it meets monitoring goals. Robust implementation would include, at a minimum: (1) using stream or pesticide application data to identify times of peak exposure, (2) validating a biological response with relevant biomarkers and (3) planning in situ exposures for when the effects of confounding variables are as low as possible. The latter could be determined by field measurement and/or published species tolerances, and will vary with context. Dissolved oxygen in our streams, for example, was not a significant driver of *Hyalella* endpoints because amphipods are tolerant of hypoxia (Irving et al., 2004). However, dissolved oxygen would very likely confound caging in streams with high organic loading and hypoxia-sensitive indicator species.

## Conclusions

Our findings urge a greater awareness of endpoint confounding by water quality variables and the need to refine sampling strategies to overcome it in biological monitoring and assessment. We conclude that streamlining and improved accuracy of monitoring is possible by focusing effort on times that impacts are more likely to be detected and attributed to contaminants of interest. While the strategy indicates only maximum potential risk, it may provide valuable ecotoxicological data where resources are limited.

## Supplementary Information

Below is the link to the electronic supplementary material.

Supplementary file1 (DOCX 70.0 KB)

## Data Availability

The datasets generated and analyzed in this study are available from the corresponding author upon request.

## Declarations

All authors have read, understood, and have complied as applicable with the statement on "Ethical responsibilities of Authors".
