# Supplementary material for: Overcoming water quality effects in biological monitoring: a case study of amphipod in situ exposures in Ontario agricultural streams
Source: Environ Monit Assess. 2025 Feb 4;197(3):239. doi: 10.1007/s10661-025-13665-8 (PMC11794351; doi:10.1007/s10661-025-13665-8)
Supplement: Supplementary file 1 — Supplementary file1 (DOCX 70.0 KB) [file 10661_2025_13665_MOESM1_ESM.docx]

**Supporting information for:** *Overcoming water quality effects in biological monitoring: A case study of amphipod in situ exposures in Ontario agricultural streams*

**Table S1.** Geographic Information and Land-use Information for *Hyalella* Caging Sites. Further information on 2005-2006 sites may be found in Bartlett et al. (2016).

| **Watershed** | **Site** | **Latitude (N) / Longitude (W)** | **Years sampled** | **Watershed description** |
| --- | --- | --- | --- | --- |
| Prudhomme Creek (PR) | Vineland | 43°11'30.4"  79°23'37.5" | 2005-2006 & 2008-2010 | Mainly agricultural with orchards and vineyards. Crossed by large transportation corridor. Partially urbanized. |
| Twenty-Mile Creek (TM) | Bailey | 43°09'09.7"  79°22'25.5" | 2005-2006 & 2009-2010 | Mainly agricultural for corn,  wheat, cereal hay, and livestock. Orchards, vineyards, and  greenhouses in lower reaches. Scattered urbanization throughout. |
|  | Cherry | 43°07.388'  79°24.355' | 2005-2006 |  |
|  | DSmithville | 43°04.704'  79°30.631' | 2005-2006 |  |
|  | USmithville | 43°06.519'  79°33.856' | 2005-2006 |  |
| Spencer Creek (SP) | Spencer | 43°16'58.1"  80°03'11.1" | 2008-2010 | Agricultural row crop with low density settlement. Natural forest and wetlands in conservation & farming areas. Recreational areas including golf courses. |
|  | Valens | 43°22.945'  80°07.884' | 2006 |  |
|  | Fourth | 43°18.011'  80°03.953' | 2006 |  |
|  | Sodom | 43°17.366'  80°03.979' | 2006 |  |
| Indian Creek (I) | Indian | 43°18'58.2"  79°48'39.0" | 2008-2010 | Mainly urban and periurban with limited row-crop agriculture and forest cover. Large transportation corridor. |
| Two-Mile Creek (2M) | Two-Mile | 43°14'48.7"  79°05'24.1" | 2008-2010 | Agricultural with vineyards, orchards, and row crops. Limited urbanization upstream of monitoring site. |
| Richardson’s (R) | Richardsons | 43°10'33.8"  79°17'11.7" | 2009-2010 | Field and greenhouse agriculture for fruit and row crops. Adjacent to large highway and medium-density settlement. |

**Table S2.** Summary of Sampling Coverage and Methodology for All *Hyalella in situ* Exposure Sites (2005-2006, 2008-2010). See Figure 1 of the main text and Table S1 for geographical and watershed characteristics. ‘Pesticides monitored’ refers to groups of compounds typically measured within the noted study period: *OP* – organophosphates, *AH* – acid herbicides, *NH* – neutral herbicides, *C* – carbamates and *O* – Other (sulfonylurea pesticides and metalaxyl). See Table S3 for specific pesticides and years of measurement.

| **Watershed/**  **Site** | **Years biomonitored** | ***In situ* exposure events (#)** | **Exposure duration (weeks)** | **Replicate cages (#)** | **Amphipods per cage (#)** | **Age at deployment (weeks)** | **Pesticides monitored (2005-2006)** | **Pesticides monitored (2008-2010)** |
| --- | --- | --- | --- | --- | --- | --- | --- | --- |
| PR-Vineland | 5 | 25 | 1 | 5 | 20 | 5 - 13 | OP, AH, NH | OP, AH, NH, C, O |
| TM-Cherry | 2 | 9 | 1 | 5 | 20 except July 2005 (N =10) | 5 - 11 | OP, AH, NH | - |
| TM-Bailey | 4 | 20 | 1 | 5 | 20 except July 2005 (N =10) | 5 - 11 | OP, AH, NH | OP, AH, NH, C, O |
| TM-DSmithville | 2 | 9 | 1 | 5 | 20 except July 2005 (N =10) | 5 - 11 | OP, AH, NH | - |
| TM-USmithville | 2 | 9 | 1 | 5 | 20 except July 2005 (N =10) | 5 - 11 | OP, AH, NH | - |
| SP-Spencer | 3 | 17 | 1 | 5 | 20 | 5 - 13 | - | OP, AH, NH, C, O |
| SP-Valens | 1 | 3 | 1 | 5 | 20 | 5 - 10 | OP, AH, NH | - |
| SP-4th Conc. | 1 | 3 | 1 | 5 | 20 | 5 - 10 | OP, AH, NH | - |
| SP-Sodom | 1 | 3 | 1 | 5 | 20 | 5 - 10 | OP, AH, NH | - |
| I-Indian | 2 | 16 | 1 | 5 | 20 | 5 - 13 | - | OP, AH, NH, C, O |
| 2M-Two-Mile | 3 | 17 | 1 | 5 | 20 | 5 - 13 | - | OP, AH, NH, C, O |
| R-Richardsons | 2 | 10 | 1 | 5 | 20 | 5 - 10 | - | OP, AH, NH, C, O |

**Table S3.** Maximum and Median Concentrations of Pesticides Detected in Study Streams, 2005-06 and 2008-10. Only compounds with US EPA Aquatic Life Benchmarks for acute effects in freshwater invertebrate are reported, with benchmark-exceeding compounds marked with an asterisk (*). Concentrations include samples taken outside of caging periods. Where Method Detection Limits (MDLs) are ranges, they reflect generally improving detection over time.

| **Type** | **Compound** | **Years Sampled** | **Detection frequency (%)** | | **Benchmark (ng/L)** | **Maximum concentration (ng/L)** | **Median concentration (ng/L)** | **MDL**  **(ng/L)** |
| --- | --- | --- | --- | --- | --- | --- | --- | --- |
| Acid | 2,4-D | All | 76 | 1.25 x 10^7^ | | 4220 | 21.1 | 0.47 – 1.73 |
| herbicides | 2,4-DB | All | 1 | 1.25 x 10^8^ | | 19.3 | 0 | 0.53 – 1.45 |
|  | 2,4-DP | All | 17 | 2.79 x 10^8^ | | 201 | 0 | 0.42 – 1.07 |
|  | Bromoxynil | All | 20 | 7.96 x 10^6^ | | 251 | 0 | 0.99 – 1.33 |
|  | Clopyralid | All | 32 | 1.17 x 10^8^ | | 88.2 | 0 | 0.59 – 1.06 |
|  | Dicamba | All | 70 | 5.0 x 10^7^ | | 831 | 3.76 | 0.73– 0.89 |
|  | MCPA | All | 25 | 4.1 x 10^7^ | | 87.5 | 0 | 0.58 – 1.32 |
|  | MCPB | All | 1 | 2.51 x 10^7^ | | 2.94 | 0 | 0.63 – 1.72 |
|  | Mecoprop | All | 79 | 4.55 x 10^7^ | | 3610 | 15.7 | 0.5 – 1.02 |
| Carbamates | Carbaryl* | 2008-10 | 70 | 850 | | 949 | 1.32 | 0.16 |
|  | Carbofuran | 2008-10 | 20 | 1115 | | 20.2 | 0 | 0.24 |
|  | Methomyl | 2008-10 | 10 | 4400 | | 6.41 | 0 | 0.30 |
|  | Oxamyl | 2008-10 | 15 | 90000 | | 292 | 0 | 0.18 |
|  | Pirimicarb | 2008-10 | 46 | 9500 | | 16.4 | 0 | 0.24 |
| Neutral | Atrazine | All | 94 | 3.6 x 10^5^ | | 6630 | 38.75 | 1.86 - 5.76 |
| herbicides | Metolachlor | All | 73 | 1.18 x 10^7^ | | 5160 | 24.1 | 1.73 - 23.7 |
|  | Metribuzin | All | 11 | 2.1 x 10^6^ | | 166 | 0 | 6.16 - 20.7 |
|  | Simazine | All | 41 | 5.0 x 10^5^ | | 2830 | 0 | 5.10 - 16.40 |
|  | Trifluralin | All | 1 | 1.26 x 10^5^ | | 19.5 | 0 | 2.66 - 5.15 |
| Organo- | Azinphos-methyl* | All | 12 | 80 | | 12200 | 0 | 10 - 138 |
| phosphates | Chlorpyrifos* | All | 20 | 7 | | 349 | 0 | 2.2 - 14.7 |
|  | Diazinon* | All | 12 | 105 | | 1650 | 0 | 13.2- 15.5 |
|  | Dimethoate | All | 3 | 21500 | | 216 | 0 | 18.1 - 25.1 |
|  | Disulfoton | All | 1 | 1950 | | 58.6 | 0 | 8.3 - 47.1 |
|  | Malathion* | All | 4 | 49 | | 611 | 0 | 3.99 - 14.7 |
|  | Parathion | All | 0.5 | 485 | | 68.3 | 0 | 4.6 - 15.5 |
|  | Phosmet | All | 0.5 | 4320 | | 10.3 | 0 | 3.66 - 157 |
|  |  |  |  |  | |  |  |  |
| **Table S3 (cont.).** | | | | | | | | |
| **Type** | **Compound** | **Years Sampled** | **Detection frequency (%)** | **Benchmark (ng/L)** | | **Maximum concentration (ng/L)** | **Median concentration (ng/L)** | **MDL**  **(ng/L)** |
| Other | Acifluorfen | 2008-10 | 1 | 1.41 x 10^7^ | | 8.78 | 0 | 5.27 - 33.3 |
|  | Clomazone | 2008-10 | 1 | 2.7 x 10^6^ | | 2.83 | 0 | 0.94 - 2.27 |
|  | Diuron | 2008-10 | 61 | 87500 | | 902 | 9.935 | 3.99 |
|  | Flumetsulam | 2008-10 | 15 | 1.27 x 10^8^ | | 100 | 0 | 0.66 - 2.07 |
|  | Fomesafen | 2008-10 | 22 | 1.88 x 10^8^ | | 792 | 0 | 2.54 - 15.4 |
|  | Foramsulfuron | 2008-10 | 2 | 5.13 x 10^7^ | | 13.65 | 0 | 9.44 |
|  | Linuron | 2008-10 | 10 | 60000 | | 145 | 0 | 9.99 - 20.2 |
|  | Metalaxyl | 2008-10 | 81 | 1.4 x 10^7^ | | 1330 | 4 | 0.42 |
|  | Metsulfuron-methyl | 2008-10 | 1 | 7.5 x 10^7^ | | 4.16 | 0 | 1.18 – 3.45 |
|  | Nicosulfuron | 2008-10 | 2 | 5.0 x 10^8^ | | 85.2 | 0 | 7.53 – 7.57 |
|  | Thifensulfuron-methyl | 2008-10 | 4 | 5.0 x 10^8^ | | 4.67 | 0 | 1.22 – 1.72 |

**Table S4.** General Linear Model Results of Caged *Hyalella* Endpoints Versus Pesticide Toxicity Index (PTI) for southern Ontario Study Streams. Data include caging events for which all pesticide types were measured. Significant terms are bolded.

| **Endpoint** | **Term** | **R^2^ _adj._** | **df** | **F** | **p** | **Semipartial correlation** |
| --- | --- | --- | --- | --- | --- | --- |
| *Mortality (%)^†^* | *Model* | *0.34* | *48* | *6.45* | *0.0001* |  |
|  | **Intercept** |  | **1** | **44.7** | **<0.0001** |  |
|  | **Organophosphate PTI^†^** |  | **1** | **4.85** | **0.033** | **0.25** |
|  | **Carbamate PTI^†^** |  | **1** | **11.28** | **0.002** | **0.38** |
|  | Acid herb. PTI^†^ |  | 1 | 2.28 | 0.138 | -0.17 |
|  | Neutral herb. PTI^†^ |  | 1 | 0.03 | 0.870 | -0.02 |
|  | Other PTI^†^ |  | 1 | 0.49 | 0.487 | -0.08 |
| *AChE inhibition (%)^†^* | *Model* | *0.10* | *45* | *2.13* | *0.079* |  |
|  | **Intercept** |  | **1** | **1018.9** | **<0.0001** |  |
|  | Organophosphate PTI^†^ |  | 1 | 3.49 | 0.068 | 0.25 |
|  | Carbamate PTI^†^ |  | 1 | 0.75 | 0.391 | 0.12 |
|  | Acid herb. PTI^†^ |  | 1 | 2.41 | 0.127 | -0.21 |
|  | Neutral herb. PTI^†^ |  | 1 | 1.16 | 0.287 | 0.14 |
|  | Other PTI^†^ |  | 1 | 0.72 | 0.400 | 0.13 |
| *Growth reduction (%)* | *Model* | *0.25* | *45* | *4.35* | *0.003* |  |
|  | **Intercept** |  | **1** | **78.12** | **<0.0001** |  |
|  | Organophosphate PTI |  | 1 | 3.69 | 0.061 | 0.24 |
|  | **Carbamate PTI** |  | **1** | **11.33** | **0.002** | **0.41** |
|  | Acid herb. PTI^†^ |  | 1 | 1.01 | 0.32 | 0.12 |
|  | **Neutral herb. PTI^†^** |  | **1** | **8.05** | **0.007** | **-0.35** |
|  | Other PTI^†^ |  | 1 | 1.29 | 0.264 | -0.14 |

^†^ Fourth-root transformed

**Table S5.** General Linear Model Results of *Hyalella* Endpoints Versus Organophosphate PTI and Water Quality Variables for southern Ontario Study Streams (2005-06, 2008-10). Significant terms are bolded.

| **Endpoint** | **Term** | **R^2^ _adj._** | **df** | **F** | **p** | **Semipartial correlation** |
| --- | --- | --- | --- | --- | --- | --- |
| *Mortality (%)^†^* | *Model* | *0.45* | *96* | *17.48* | *<0.0001* |  |
|  | Intercept |  | 1 | 0.87 | 0.355 |  |
|  | **Organophosphate PTI^†^** |  | **1** | **23.51** | **<0.0001** | **0.36** |
|  | **Mean temperature^‡^** |  | **1** | **25.22** | **<0.0001** | **0.37** |
|  | Mean dissolved oxygen |  | 1 | 1.84 | 0.179 | -0.17 |
|  | Mean pH |  | 1 | 2.03 | 0.158 | -0.02 |
|  | Mean conductivity |  | 1 | 0.63 | 0.431 | -0.08 |
| *AChE inhibition (%)^†^* | *Model* | *0.18* | *86* | *4.92* | *0.0005* |  |
|  | **Intercept** |  | **1** | **40.95** | **0** |  |
|  | **Organophosphate PTI^†^** |  | **1** | **25.73** | **0** | **0.46** |
|  | Mean temperature^‡^ |  | 1 | 3.03 | 0.085 | 0.17 |
|  | Mean dissolved oxygen |  | 1 | 0.81 | 0.37 | 0.09 |
|  | Mean pH |  | 1 | 0.58 | 0.447 | 0.07 |
|  | Mean conductivity |  | 1 | 2.41 | 0.124 | -0.15 |
| *Growth reduction (%)* | *Model* | *0.15* | *82* | *4.07* | *0.002* |  |
|  | **Intercept** |  | **1** | **4.25** | **0.043** |  |
|  | **Organophosphate PTI^†^** |  | **1** | **3.96** | **0.050** | **0.20** |
|  | **Mean temperature** |  | **1** | **7.75** | **0.007** | **-0.28** |
|  | Mean dissolved oxygen |  | 1 | 0.53 | 0.469 | -0.01 |
|  | Mean pH |  | 1 | 0.01 | 0.915 | -0.01 |
|  | Mean conductivity |  | 1 | 0.68 | 0.412 | 0.08 |

^†^ Fourth-root transformed

^‡^ Log transformed

**Table S6.** Alternative Linear Model of *Hyalella* Endpoints Versus Organophosphate PTI, Water Quality Variables and Temperature-Pesticide Interaction. Only endpoints significantly influenced by temperature and organophosphate toxicity (see Table S5) are reported. Significant terms are bolded.

| **Endpoint** | **Term** | **R^2^ _adj._** | **df** | **F** | **p** | **Semipartial correlation** |
| --- | --- | --- | --- | --- | --- | --- |
| *Mortality (%)^†^* | *Model* | *0.52* | *95* | *17.10* | *<0.0001* |  |
|  | Intercept |  | 1 | 1.94 | 0.355 |  |
|  | **Organophosphate PTI^†^** |  | **1** | **5.22** | **0.025** | **-0.16** |
|  | **Mean temperature^‡^** |  | **1** | **16.83** | **0** | **0.29** |
|  | Mean dissolved oxygen |  | 1 | 0.98 | 0.324 | -0.07 |
|  | Mean pH |  | 1 | 2.68 | 0.105 | -0.12 |
|  | Mean conductivity |  | 1 | 0.87 | 0.354 | 0.07 |
|  | **Organophosphate PTI^†^ x Mean temperature^‡^** |  | **1** | **8.44** | **0.005** | **0.21** |
| *Growth reduction (%)* | *Model* | *0.15* | *81* | *4.55* | *0.0005* |  |
|  | **Intercept** |  | **1** | **4.12** | **0.046** |  |
|  | Organophosphate PTI^†^ |  | 1 | 2.95 | 0.09 | -0.17 |
|  | **Mean temperature** |  | **1** | **10.33** | **0.002** | **-0.31** |
|  | Mean dissolved oxygen |  | 1 | 0.06 | 0.809 | -0.02 |
|  | Mean pH |  | 1 | 0.02 | 0.913 | -0.01 |
|  | Mean conductivity |  | 1 | 1.03 | 0.313 | 0.10 |
|  | **Organophosphate PTI^†^ x Mean temperature** |  | **1** | **5.74** | **0.019** | **0.23** |

^†^ Fourth-root transformed

^‡^ Log transformed

**Table S7.** Alternative Linear Model Results of *Hyalella* Endpoints Versus Organophosphate and Carbamate PTIs and Water Quality Variables for southern Ontario Study Streams. Because carbamates were only sampled from 2008 to 2010, analyses are restricted to these dates. Significant terms are bolded.

| **Endpoint** | **Term** | **R^2^ _adj._** | **df** | **F** | **p** | **Semipartial correlation** |
| --- | --- | --- | --- | --- | --- | --- |
| *Mortality (%)^†^* | *Model* | *0.42* | *52* | *8.01* | *<0.0001* |  |
|  | Intercept |  | 1 | 1.45 | 0.235 |  |
|  | **Organophosphate PTI^†^** |  | **1** | **8.43** | **0.005** | **0.29** |
|  | **Carbamate PTI^†^** |  | **1** | **4.44** | **0.039** | **0.21** |
|  | **Mean temperature^‡^** |  | **1** | **9.16** | **0.004** | **0.3** |
|  | Mean dissolved oxygen |  | 1 | 0.63 | 0.432 | 0.08 |
|  | Mean pH |  | 1 | 2.95 | 0.092 | -0.17 |
|  | Mean conductivity |  | 1 | 0 | 0.986 | 0 |
| *AChE inhibition (%)^†^* | *Model* | *0.25* | *48* | *3.95* | *0.003* |  |
|  | **Intercept** |  | **1** | **11.43** | **0.001** |  |
|  | **Organophosphate PTI^†^** |  | **1** | **6.16** | **0.014** | **0.29** |
|  | **Carbamate PTI^†^** |  | **1** | **5.86** | **0.019** | **0.29** |
|  | Mean temperature^‡^ |  | 1 | 1.4 | 0.243 | 0.14 |
|  | **Mean dissolved oxygen** |  | **1** | **9.25** | **0.004** | **0.36** |
|  | Mean pH |  | 1 | 0.55 | 0.461 | -0.09 |
|  | **Mean conductivity** |  | **1** | **11.44** | **0.001** | **-0.40** |
| *Growth reduction (%)* | *Model* | *0.22* | *48* | *3.57* | *0.005* |  |
|  | **Intercept** |  | **1** | **8.34** | **0.006** |  |
|  | Organophosphate PTI |  | 1 | 0.22 | 0.64 | 0.07 |
|  | **Carbamate PTI** |  | **1** | **6.21** | **0.016** | **0.30** |
|  | **Mean temperature** |  | **1** | **5.88** | **0.019** | **-0.29** |
|  | Mean dissolved oxygen |  | 1 | 0.06 | 0.81 | 0.03 |
|  | Mean pH |  | 1 | 3.2 | 0.08 | -0.21 |
|  | Mean conductivity |  | 1 | 1.17 | 0.285 | 0.13 |

^†^ Fourth-root transformed

^‡^ Log transformed

**Table S8.** Decision Matrix for Optimizing *in situ Hyalella* Caging During Agricultural Season. Optimal caging for gauging maximum risk is at times of (i) high pesticide exposure, (ii) high biomarker (AChE Inhibition) response indicating exposure and (iii) limited confounding of endpoints (e.g., mortality) by water quality variables (e.g., temperature).

| **Caging Month** | **Pesticide exposure** | **Biomarker Response** | **Potential for Endpoint Confounding** |
| --- | --- | --- | --- |
| April | Medium | Medium | Low |
| May | Medium | High | Low |
| June | High | High | Medium |
| July | Medium | High | High |
| August | Medium | Medium | High |
| September | Low | Medium | Medium |
| October | Low | Low | Low |

**Table S9.** Median Values of Organophosphate PTI, Endpoint and Biomarker Responses for Reference and High-Impact Sites, 2010-2010. Test statistics and p-values for Kruskal-Wallis ANOVA & Median Test are reported. *a* and *b* superscripts denote groups indicated by post-hoc comparisons (for significant Median Tests only, highlighted in bold).

| **Variable** | **Caging month** | **χ^2^** | **p** | **Indian Creek** | | **Spencer Creek** | **Two-Mile Creek** | **Prudhomme Creek** |
| --- | --- | --- | --- | --- | --- | --- | --- | --- |
| Organophosphate PTI | **All** | **32.17** | **<0.0001** | | **0^a^** | **0^a^** | **1.9^b^** | **2.43^b^** |
| Mortality (%) | April | 4.00 | 0.262 | | 5 | 0 | 0 | 5 |
|  | May | 5.60 | 0.130 | | 2.5 | 5 | 0 | 5 |
|  | **June** | **36.53** | **<0.0001** | | **5^a^** | **5^a^** | **100^b^** | **95^b^** |
|  | **July** | **23.73** | **<0.0001** | | **15^a^** | **10^a^** | **100^b^** | **15^a^** |
|  | **August** | **21.96** | **0.0001** | | **65^a^** | **5^b^** | **90^a^** | **10^ab^** |
|  | **September** | **12.80** | **0.005** | | **37.5^ab^** | **5^a^** | **100^b^** | **25^ab^** |
|  | **October** | **21.54** | **0.001** | | **5^ab^** | **0^a^** | **25^b^** | **10^b^** |
| AChE Inhibition (%) | **April** | **13.60** | **0.004** | | **11.0^a^** | **37.8^ab^** | **20.9^ab^** | **55.4^b^** |
|  | **May** | **14.40** | **0.002** | | **27.0^a^** | **22.9^ab^** | **15.9^b^** | **38.6^a^** |
|  | **June** | **22.02** | **0.0001** | | **18.1^a^** | **27.0^a^** | **52.0^ab^** | **74.7^b^** |
|  | **July** | **11.90** | **0.008** | | **16.3^a^** | **24.8^ab^** | **43.7^b^** | **34.2^b^** |
|  | August | 7.14 | 0.067 | | 12.5 | 21.6 | 16.1 | 42.0 |
|  | September | 4.80 | 0.187 | | 19.3 | 23.5 | -2.1 | 18.3 |
|  | **October** | **8.80** | **0.032** | | **2.1^a^** | **14.8^b^** | **11.3^ab^** | **10.3^ab^** |
| Growth reduction | April | 0.80 | 0.850 | | -12.0 | -5.7 | -3.2 | -4.1 |
| (%) | May | 3.20 | 0.362 | | -2.3 | -4.5 | -1.9 | -2.9 |
|  | **June** | **11.34** | **0.010** | | **-6.7^ab^** | **-18.4^a^** | **35.9^b^** | **11.8^b^** |
|  | **July** | **8.94** | **0.030** | | **-3.7^ab^** | **-11.4^a^** | **5.6^b^** | **-2.8^ab^** |
|  | **August** | **12.58** | **0.006** | | **-9.9^a^** | **-8.9^a^** | **5.6^a^** | **2.3^a^** |
|  | September | 6.40 | 0.094 | | -1.4 | -2.1 | 21.6 | 3.4 |
|  | October | 0.27 | 0.966 | | -4.3 | -3.1 | -1.0 | -2.4 |

**Table S10.** Results of Discriminant Analysis Differentiating Two Site Categories of Reference (Spencer and Indian Creeks) and High-Impact (Prudhomme and Two-Mile Creeks) Sites in Different Caging Months, 2008-2010.

| **Caging Month** | **F** | **p** | **Significant variables** | **Cases correctly assigned (%)** |
| --- | --- | --- | --- | --- |
| All months | 23.73 | <0.0001 | Mortality^†^, AChE inhibition,  growth reduction | 75.0 |
| April | 1.28 | 0.314 | - | 60.0 |
| May | 0.48 | 0.700 | - | 60.0 |
| June | 50.95 | <0.0001 | Mortality^†^, AChE inhibition,  growth reduction | 100.0 |
| July | 10.30 | 0.0001 | Mortality^†^, AChE inhibition | 77.1 |
| August | 5.48 | 0.006 | AChE inhibition,  growth reduction | 70.4 |
| September | 4.71 | 0.008 | Growth reduction | 76.5 |
| October | 7.21 | <0.001 | Mortality^†^ | 71.7 |

^†^ Fourth-root transformed
